# Determination of cut-off and correlates of delay in treatment-seeking of febrile illness: a retrospective analysis

**DOI:** 10.1186/s12889-020-08660-2

**Published:** 2020-04-28

**Authors:** Himanshu K. Chaturvedi, Ram C. Bajpai, Preeti Tiwari

**Affiliations:** 1 ICMR-National Institute of Medical Statistics, Indian Council of Medical Research, Ansari Nagar, New Delhi, 110 029 India; 2 School of Primary, Community and Social Care, Keele University, Staffordshire, ST5 5BG UK

**Keywords:** Delay in reporting, Febrile illness, Malaria, Cut-off for the delay, Northeast India

## Abstract

****Background**:**

Early diagnosis and treatment of malaria symptoms reduces the risk of severe complication and malaria transmission. However, delay in malaria diagnosis and treatment is a major public health problem in India. The primary aim of the study was to determine cut-off for the delay in seeking treatment of fever, and the secondary aim was to identify the factors associated with delay in malaria-endemic areas of Assam, Northeast India.

****Methods**:**

The present study analysed data from two prior cross-sectional surveys (community- and hospital-based) that was conducted to study the health-seeking behaviour of people residing in high malaria-endemic areas of Assam, Northeast India. The hospital-based survey data were used to determine optimal cut-off for the delay in reporting, and further, used to identify the factors associated with delay using community-based data.

****Results**:**

Mean age of fever cases was similar in both community- and hospital-based surveys (23.1 years vs 24.2 years, *p* = 0.229). Delay in reporting fever was significantly higher among hospital inpatients compared to community-based fever cases (3.6 ± 2.1 vs 4.0 ± 2.6 days; *p* = 0.006). Delay of > 2 days showed higher predictive ability (sensitivity: 96.4%, and ROC area: 67.5%) compared to other cut-off values (> 3, > 4, and > 5 days). Multivariable logistic regression analysis revealed that the adjusted odds ratio (aOR) of delay was significantly higher for people living in rural areas (1.52, 95%CI: 1.11–2.09), distance (> 5 km) to health facility (1.93, 95%CI: 1.44–2.61), engaged in agriculture work (2.58, 95%CI: 1.97–3.37), and interaction effect of adult male aged 20–40 years (1.71, 95%CI: 1.06–2.75).

****Conclusion**:**

The delay (> 2 days) in seeking treatment was likely to be twice among those who live in rural areas and travel > 5 km to assess health care facility. The findings of the study are useful in designing effective intervention programmes for early treatment of febrile illness to control malaria.

## Background

The World Health Organization (WHO) has accentuated that early diagnosis and prompt treatment for malaria should be occurred within 24–48 h of the onset of malaria symptoms to decrease the risk of severe complications and onward transmission [1]. Good treatment-seeking behaviour and easy access to health services are important components imperative to its success. A prior study recommended that patients should seek medical treatment following the onset of fever, a common symptom of malaria [2]. It has been recognised that self-treatment may lead to more delay in seeking treatment [2, 3]. Such delay may cause severe complications to patient within 3–7 days of onset of fever. The *Plasmodium falciparum* malaria causes a delay in cure, severe disease or death especially in multi-drug resistance areas [4].

Fever, the most common symptom of malaria, can be intermittent or continuous accompanied with other symptoms such as chills and rigours, headache, myalgia, arthralgia, anorexia, nausea and vomiting. The symptoms of malaria can be non-specific and mimic other diseases such as viral infections, and enteric fever etc. [5]. All fever cases are diagnosed as malaria either by rapid diagnostic kit (RDT) or microscopy, and choice of medicine depends upon whether the patient has *P. vivax* or *P. falciparum* malaria [6].

India has the highest number of malaria cases and related deaths in the Southeast Asia region [7, 8]. According to the National Vector Borne Disease Control Program (NVBDCP) annual report (2014–15), about 91% of malaria cases and 99% of deaths due to malaria was reported from high disease burden states namely North-eastern (NE) States, Andhra Pradesh, Chhattisgarh, Gujarat, Jharkhand, Karnataka, Madhya Pradesh, Maharashtra, Odisha, Rajasthan and West Bengal [9]. High malaria transmission in Northeast India was due to the presence of various malaria parasites and vector species. The ecological condition, high rainfall and humid climate are favourable to growth and proliferation of the parasites and vectors in this region [9]. A previous study from Assam showed only 43% of febrile illness cases utilised government/private health services, and remaining used traditional and/or self-medication [3]. The consequence of traditional and self-medication could result in misdiagnosis and incorrect choice of drugs, delay in diagnosis of malaria and increasing malaria transmission in the community [2, 3]. The poor health care utilisation and orthodox health beliefs such as go-to priest, perform spiritual prayers, sacrifice a bird/animal are the key obstacles to early diagnosis of febrile illness [2, 3].

Malaria is curable with early diagnosis and treatment. Delay in treatment can lead to profound consequences including death [1, 4]. An integrated approach of comprising both prevention and treatment with effective antimalarial agents is required to control Malaria. Prompt and effective treatment is also important for controlling the transmission of malaria [1]. Thus, to avoid such terrible effect of malaria it is important to catalyse the delay to treatment from the onset of fever and improve health-seeking behaviour of people [2].

Early treatment of febrile illness has been emphasised in the literature to control malaria, however, the knowledge about the correlates of the delay in seeking treatment is limited and without any defined cut-off for the delay. The optimum cut-off for the delay in the treatment of febrile illness is also required to work out especially for the malaria-endemic areas. In this study, we aimed to determine the optimum cut-off for the delay in seeking treatment of febrile illness and identify the factors associated with delay in the malaria-endemic areas of Assam, Northeast India.

## Methods

### Study area

**Fig. 1:**
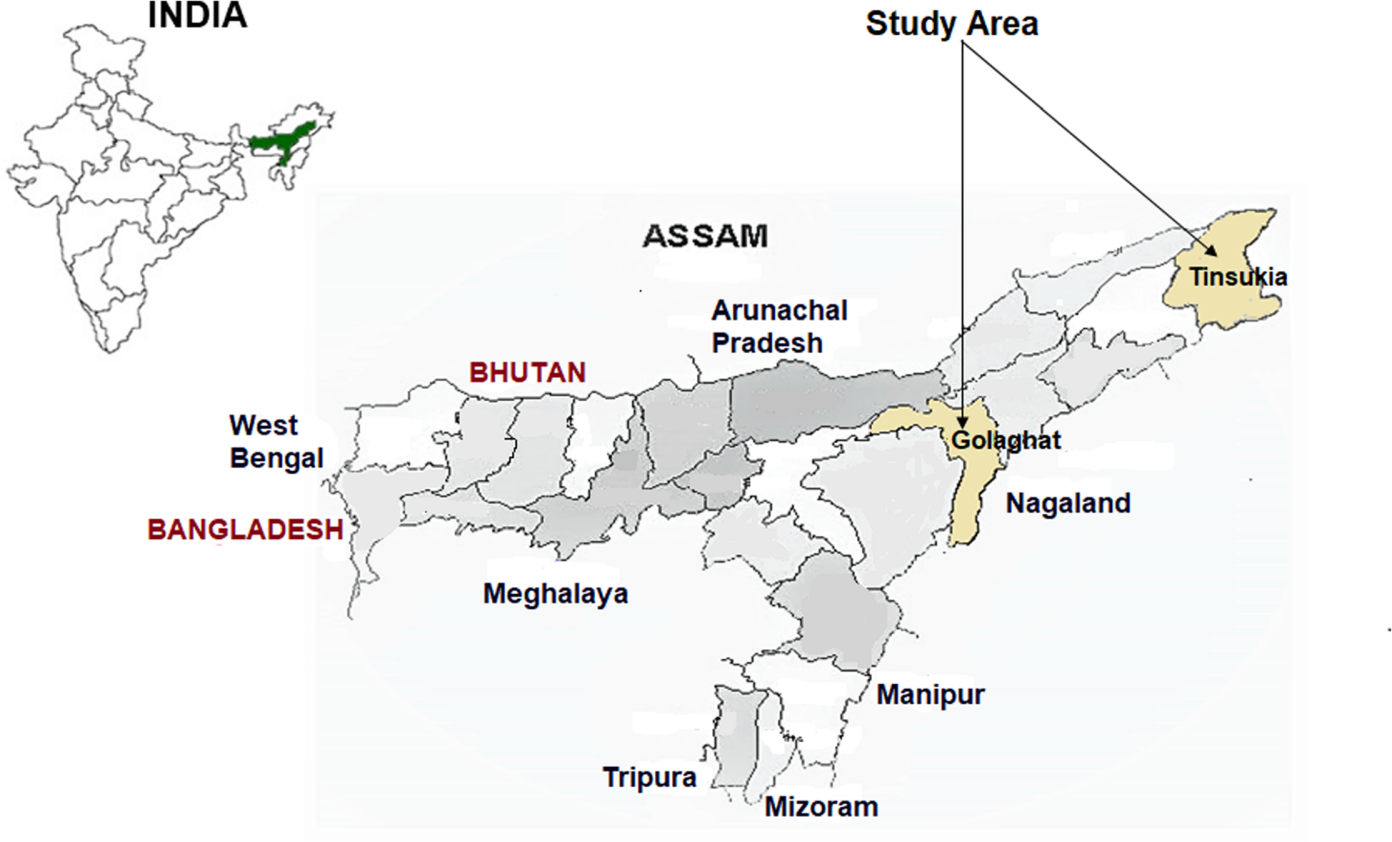
The map shows the geographic location of Tinsukia and Golaghat districts of Assam selected as study area. Source: http://censusindia.gov.in/maps/State_Maps/StateMaps_links/assam.jpg

Assam is bordered by Bhutan and Arunachal Pradesh to the north; Nagaland and Manipur to the east; Meghalaya, Tripura, Mizoram and Bangladesh to the south; and West Bengal to the west (Fig. 1). The state has total population 31.17 million with geographical area 78,438 km^2^ and thus the population density 398 persons per km^2^; and 85.9% population shared by the rural region of Assam (Census 2011). Production of tea, crude oil, natural gas, silk etc. are the major economic resources of the state. Assam, a flood-prone area, constitutes two major valleys namely Brahmaputra and Barak which intersperses hill ranges, difficult terrain and an evergreen rain forest covering nearly 40% of the geographical area. With pre-monsoon showers (March and April), the heavy rainfall of two meters or more recorded during monsoon from July to September. The relative humidity varying from 70 to 85% throughout the year makes the overall environment conducive for mosquito proliferation, survival, and longevity and favours active malaria transmission. Health services in the state were mainly provided by the government (district hospitals, community health centres (CHCs), primary health centres (PHCs), sub-centres (SHCs)) and private service providers (private hospitals, tea-garden hospitals, other hospitals of industries). Health insurance schemes for the general public and cashless health insurance scheme by the government for poor people has been started, but its coverage and impact on public health is not yet known. A study on health-seeking behaviour of people was conducted in two districts of high malaria-endemic area of upper Assam, namely Tinsukia and Golaghat. Data were collected at the household level to assess the treatment-seeking behaviour of people and at the health facility level to know the prevalence of malaria among febrile patients.

### Study design

We used data from two previously conducted surveys on health-seeking behaviour of febrile illness of people in Assam state of India. One survey was conducted among inpatients in some pre-selected hospitals, whereas the other survey was conducted at the household level in the same selected two districts during the same period. Hospital-based survey data were used to find the optimal cut-off for the delay in reporting to the hospital for seeking treatment and risk of malaria among them, whereas household survey data were used to apply the obtained cut-off to know the factors associated with delay. A brief description of hospital- and community-based surveys is presented below.

#### Community-based survey

Household survey was carried out in randomly selected 100 primary sampling units (PSUs) covering wards in urban and villages in rural areas of the selected study area. In each PSU’s, a list of all households was prepared and those households who had reported a case of malaria or fever in last 3 months and described the symptom of disease before seeking treatment were recorded. A sample of 20 such households who received treatment of fever was selected randomly. If the list of such households was less than or equal to 20, all of them were included for the survey. The head of the selected households was interviewed to collect general information whereas specific information related to the treatment of fever was collected from the individual patient or mother of sick child in a pre-tested questionnaire [3].

#### Hospital-based survey

Survey was also conducted in six government hospitals (three in urban and three in rural), six private hospitals and four tea garden hospitals (i.e. community hospitals) to collect information from the inpatients reported for treatment of febrile illness. These hospitals were selected based on health facility services for the treatment of malaria and the availability of patients reported with fever. All the febrile patients who reported to the selected hospitals during the study period were assessed by the physician and those who were admitted and suspected of having malaria were included in the study. Patients admitted to the hospital with febrile illness, but who were not suspected of having malaria, were excluded. The patients included in the study were interviewed by trained Field Investigators for collection of general information related to health-seeking behaviour including delay in reporting for treatment, utilization of health services and diagnosis of fever using the pre-tested questionnaire [2].

### Data analysis

Data used for this study were extracted from household survey on health-seeking behaviour and hospital-based survey of febrile patients reported for treatment. Background characteristics such as age, sex, religion, place of residence, monthly family income, and distance to health care facility etc. were considered to assess any association with delay in seeking tratement [2, 3]. Age was categorized into three groups as < 20, 20–40 and > 40 years. Occupation of respondents was also categorized into working groups such as agriculture and non-agricultural work (self-employed and service), and not-working group (housewife and others). The family income of respondents was categorized as < 5000 and ≥ 5000 INR. The normality of the continuous variables is tested by Shapiro-Wilk and Shapiro-Francia test statistics before comparing the means by Mann-Whitney U test or Student’s t-test. The Spearman rank correlation was used to explore the correlation between delay in treatment of fever and duration of fever treatment.

We hypothesised that hospital-based fever data were collected in more controlled conditions. Therefore, we used it to find optimal cut-off for the delay in reporting (days). Number of multivariable logistic regression models were fitted for the delay in reporting at each cut-points i.e., > 2, > 3, > 4 and > 5 days respectively. Various goodness-of-fit measures were calculated for each fitted model and compared with each other to find the best fit for the fever data at a given cut-off value. Overall fit was assessed by log-likelihood, Akaike information criterion (AIC), Bayesian information criterion (BIC), and Hosmer-Lemeshow test. Model discrimination was assessed by area under the receiver operating characteristic (AUROC), and Somer’s D statistics. Model classification was measured by sensitivity, specificity, positive predictive value (PPV), negative predictive value (NPV) and the overall ability for correct classification. Model calibration was assessed by Brier score, calibration-in-the-large coefficient (CITL), expected/observed ratio, calibration slop, and calibration plot. Our decision for optimal cut-off was based on goodness-of-fit performance in the majority of indices [10–12].

Further, we validated this cut-off in the community-based fever data. We fitted univariable and multivariable logistic regression models to find factors associated with the delay in reporting. The odds ratios (ORs) were calculated and reported with corresponding 95% confidence intervals (CIs). We also checked for possible interactions between delay in reporting, and predictor variables, and reported in the regression table if its *p*-value was found to be less than 0.1 (using more conservative criteria). The goodness-of-fit for the multivariable model was ascertained by overall fit, discrimination, calibration, and classification as previously described. We used Stata 14.2 (StataCorp, College Station, TX, US) statistical software for analysis of the data.

## Results

The socio-demographic profile of fever cases including delay in reporting and duration of treatment are presented for the two surveys in Table 1. Significant differences in the pattern of distribution by age, gender, type of work related to occupation, place of residence and health services used for treatment was observed between two surveys of fever cases. The average age of fever cases was similar in both surveys (23.1 years in community and 24.2 years in hospital surveys; *p* = 0.229). The average household/family income reported was significantly higher in the hospital survey (INR 3610) compare with household survey (INR 2534). The mean delay in reporting for treatment of fever was recorded significantly higher in-hospital survey (4 days in hospital and 3.6 days in community surveys; *p* < 0.01), whereas average expenditure on medicines (Indian rupees 455 in hospital and 118 in community surveys; *p* < 0.001) was three times more for fever patients reported to hospitals. Of the 350 cases reported with febrile illness, 97 (27.7%) cases were diagnosed as malaria in the hospital survey. Distribution of delay in reporting for treatment and moderate positive correlation (Spearman’s rho = 0.54; *p* < 0.01) was observed between the delay in reporting fever and duration of treatment (Supplementary Fig. S1 & S2).

**Table 1:** General profile of fever cases in the community and hospital surveys in the malaria-endemic areas of Assam, Northeast India

Background characteristics	Type of survey		χ^**2**^ / Z test	***p***-value
	Community	Hospital		
**Total fever cases**	**1989**	**350**		
**District (*n*, %)**				
Golaghat	995 (50.0)	187 (53.4)	0.9	0.329
Tinsukia	994 (50.0)	163 (46.6)		
**Age group (years) (*n*, %)**				
< 20	1080 (54.3)	147 (42.0)	33.2	< 0.001
20–40	525 (26.4)	145 (41.1)		
> 40	383 (19.3)	58 (16.6)		
**Gender (*n*, %)**				
Female	1171 (58.9)	132 (37.7)	54	< 0.001
Male	818 (41.1)	218 (62.3)		
**Religion (*n*, %)**				
Hindu	1841 (92.6)	316 (90.3)	2.1	0.143
Others	148 (7.4)	34 (9.7)		
**Occupation (*n*, %)**				
Not-working	528 (26.5)	127 (36.3)	52.4	< 0.001
Agriculture	774 (38.9)	170 (48.6)		
Non-agriculture	687 (34.5)	53 (15.1)		
**Place of residence (*n*, %)**				
Rural	1750 (88.0)	113 (32.3)	569.6	< 0.001
Urban	239 (12.0)	237 (67.7)		
**Health Services (*n*, %)**				
Government	1303 (65.5)	198 (56.6)	106.1	< 0.001
Private	686 (34.5)	152 (43.4)		
**Continuous data (mean ± SD)**				
Age of fever cases (years)	23.1 ± 18.7	24.2 ± 15.2	1.2	0.229
Monthly family income (INR)	2534 ± 1816	3610 ± 2029	9.29	< 0.001
Distance to health centre (km)	4.2 ± 5.0	17.0 ± 20.1	11.9	< 0.001
Delay in reporting (days)	3.6 ± 2.1	4.0 ± 2.6	2.7	0.006
Duration of treatment (days)	4.5 ± 3.2	4.3 ± 3.8	0.9	0.353
Expenditure on medicines (INR)	118.1 ± 248.6	454.5 ± 388.6	15.7	< 0.001

Table 2 presents the number of goodness-of-fit measures for each fitted model using the hospital-based survey. Overall delay > 2 days cut-off showed better fit in the majority of model fitting parameters. Overall discrimination ability was highest to delay > 2 days (ROC area = 0.6748, and Somer’s D statistic = 0.3495) with compare to other cut-offs. Model classification parameters such as sensitivity, specificity, PPVs, NPVs, and overall classification also indicated good fit for delay > 2 days (Supplementary Fig. S3). The model calibration indices such as Brier scores, CITLs, and calibration slops also indicated the better fit for the delay > 2 days. Calibration slopes for each fitted model were also indicating better prediction for delay > 2 days (Supplementary Fig. S4).

**Table 2:** Goodness-of-fit measures for various cut-offs for the delay in reporting among fever cases using hospital-based survey data (*N* = 350)

Goodness-of-fit parameters	Logistic models with various cut-offs for delay			
	> 2 days	> 3 days	> 4 days	> 5 days
**Overall model fit**				
Log likelihood	− 195.8208	− 230.0087	− 207.0900	−171.8360
AIC	417.6415	486.0173	440.1800	369.6721
BIC	467.7946	536.1704	490.3331	419.8252
Hosmer-Lemeshow test p-value	0.5965	0.5136	0.4436	0.2497
**Model discrimination**				
ROC area	0.6748	0.6474	0.6626	0.6430
Somers’ D statistic	0.3495	0.2947	0.3253	0.2860
**Model classification**				
Sensitivity	0.9639	0.6222	0.1339	0.0000
Specificity	0.1485	0.6118	0.9370	100.00
Positive predictive value (PPV)	0.7362	0.6292	0.5000	0.0000
Negative predictive value (NPV)	0.6250	0.6047	0.6969	0.7914
Correctly classified	0.7286	0.6171	0.6800	0.7914
**Model calibration**				
Brier score	0.1876	0.2327	0.2031	0.1585
CITL	−2.63E-10	4.90E-08	4.95E-08	−7.06E-08
E/O ratio	1	1	1	1
C-slope	−1.79E-08	−9.34E-10	−4.49E-09	1.26E-08

*AIC* Alike information criterion, *BIC* Bayesian information criterion, *ROC* receiver operation characteristic, *CITL* calibration-in-the-large, *E/O* expected/observed, *C-slope*: calibration-slope

**Fig. 2:**
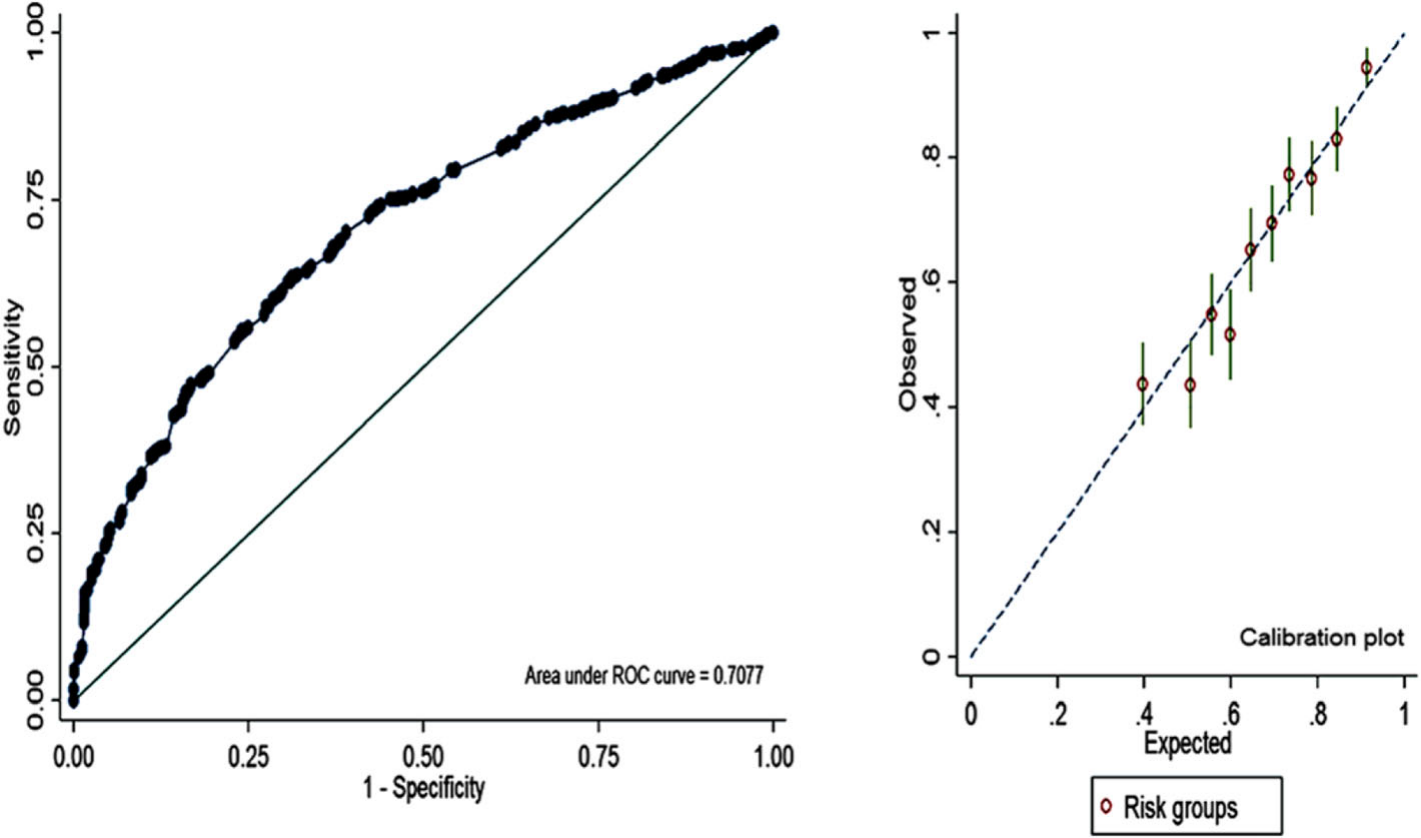
Overall model discrimination and calibration plots for the delay in reporting fever > 2 days using a community-based survey

Further, the results of univariable and multivariable logistic regression analyses for delay (> 2 days) in reporting with estimated unadjusted and adjusted odds ratio (aOR) associated with various socio-demographic factors are presented in Table 3 using community-based survey. Location, religion, type of work, place of treatment, distance to hospital and type of hospital were significantly associated with the delay in reporting fever in the community-based survey. The interaction between age group 20–40 years and male gender (aOR 1.71; *p* = 0.010) was also associated with the higher odds for the delay in reporting for treatment of malarial fever. The fitted model for community-based survey showed 86.8% sensitivity, 33.5% specificity, and 68.6% overall classification. The Somer’s D statistic was 0.415, Brier score was 0.20, CITL was − 0.033, expected/observed ratio was 1.01, and C-slope was 1.042. All of these measures were indicating good fit for delay > 2 days in the community-based survey. Overall discriminative ability and model calibration is presented in Fig. 2.

**Table 3:** Correlates of delay (> 2 days) in treatment-seeking for febrile illness among the people (community-based survey) of malaria-endemic areas of Assam, Northeast India

Characteristics	N	Delay (%)	Unadjusted OR (95% C.I.)	Adjusted OR (95% C.I.)
**District**				
Golaghat	995	61.2	1	1
Tinsukia	994	70.4	1.51f (1.25–1.82)	2.13f (1.73–2.68)
**Age group (year)**				
< 20	1080	63.4	1	1
20–40	525	66.7	1.16 (0.93–1.44)	0.85 (0.60–1.20)
> 40	383	71.5	1.45f (1.13–1.87)	1.15 (0.75–1.75)
**Gender**				
Female	1171	67.6	1.22e (1.01–1.47)	0.85 (0.65–1.11)
Male	818	63.2	1	1
**Religion**				
Hindu	1841	66.6	1.56e (1.11–2.19)	1.99f (1.36–2.92)
Others	148	56.1	1	1
**Monthly family income (INR)**				
< 5000	1772	67.2	1.68f (1.27–2.24)	1.18 (0.86–1.62)
≥ 5000	217	54.8	1	1
**Type of work**				
Not working	528	51.9	1	1
Agriculture	774	79.6	3.61f (2.83–4.61)	2.58f (1.97–3.37)
Non-agriculture	687	61.0	1.45f (1.15–1.82)	1.54f (1.20–1.96)
**Place of residence**				
Rural	1750	68.0	2.14f (1.63–2.82)	1.52e (1.11–2.09)
Urban	239	49.8	1	1
**Distance to health centre (km)**				
≤ 5	1565	61.5	1	1
> 5	424	81.6	2.77f (2.13–3.62)	1.93f (1.44–2.61)
**Type of hospital**				
Government	1303	72.6	2.36f (1.94–2.86)	2.41f (1.92–3.03)
Private	686	52.9	1	1
**Interaction terms**				
Age 20–40 years*Males				1.71e (1.06–2.75)
**Total**	**1989**	**65.8**	–	–

Note: *OR* odds ratio, *C.I*. confidence interval, *INR* Indian rupees; e = *p* < 0.05; f = *p* < 0.01

## Discussion

Delay in treatment of malaria may cause severe illness and fatality. As reported, delay in the onset of treatment and the presence of complications on admission was found to be associated with mortality [13–15]. The factors associated with the delay in reporting for the treatment were evaluated in this study. Analysis of community-based survey of 1989 fever cases and hospital-based survey of 350 patients reported with fever indicates that the delay in seeking treatment of fever was mainly associated with severity of illness, age, and gender of patients. Additionally, the type of health facilities and its distance from the village/residence have also been identified as important factors [16].

To obtain the optimum cut off for delay to malaria fever case prediction, ROC curve approach has been used which is also defined as one of the appropriate methods to obtain optimum cut-point value in any scale by some other studies [10–12]. Optimum cut off for delay in reporting of fever cases that maximize (sensitivity + specificity) for higher prediction of malaria cases was found to be at > 2 days, which also observed as optimum for malaria cases in another study [16].

Socio-demographic factors are found to be associated with delay in reporting for treatment such as age, gender, place of residence, monthly income, place of treatment, occupation, distance of health facility and type of facility. Delay in treatment-seeking was likely to be 1.5 times more among older age people (40+ years). It was also about two times more among those who engaged in agriculture occupation. Their negligence and prior commitment related to agriculture or farming work was the main reason of the delay as they were scared to lose their daily wages. In a study conducted in the Odisha state of India, the daily wage labours or small-scale farmers are not prompt to treatment-seeking [14], and such similar findings were also reported in other studies [17].

Delay in seeking treatment among rural people was likely to be two times higher as compared with urban, it was possibly due to distance and lack of proper facilities of health care services near their residence. In this study, most of the participants were resided in rural area and required to travel more than 5 km to get health facilities that could also delay seeking timely treatment, as previously reported by others [14, 16, 18–20].

Other symptoms with fever was also associated with delay and it has been described in many other studies. Mean days of the delay was slightly lower (< 4 days) in case of some symptoms such as joint pain, shivering and vomiting with fever for a community survey, otherwise, it was higher (> 4 days) with most of the symptoms with fever in community and hospital surveys. Such finding was also reported in hospital-based studies [21, 22].

Many studies reported that negligence by the patients was the primary reason for delay [14, 17, 22–26]. Similar findings are recorded in our study as 2–4 days delay in reporting for treatment of fever with other symptoms related to malaria was observed in both surveys. Delay was also possibly due to the usage of traditional remedies and consultation to local unauthorised health service providers [3, 27]. The main strength of our study is using both datasets that complement each other to draw valid conclusions. However, there are some limitations of this study as we used hospital-based data to find optimal cut-off which may not have an actual representation of the general population. Another caveat to mention that the findings are based on retrospective data analysis. We also did not know about some other factors such as local medications, connectivity by road, and surface transport, etc. which may cause a delay in treatment as these information were not collected in the surveys.

## Conclusion

Overall, the study presents valuable information related to the possible cause of delay which is useful for effective health policy and plan of a community-based intervention to eliminate malaria. Early treatment of febrile illness within 2 days of onset of fever to be implemented especially in the malaria-endemic areas. The consequences of self-medication and traditional beliefs may be discussed in the community and such practices need to be discouraged. As the distance of the health centre is a major cause of delay, the local transport or ambulance service may be provided by the government in rural areas for their convenience to commute. There is a clear need to have an integrated approach to provide minimum required health care services to the rural community in their village periphery and also to create awareness about early treatment of malaria through trained health workers. Elimination of malaria can be achieved, if health administration, private industries (tea, crude oil, and natural gas), and other local non-government organizations including local leaders of society make a joint effort for this endeavour.

## Supplementary information

**Additional file 1 Figure S1**. Spearman rank correlation between delay in reporting fever and duration of fever treatment in community-based survey (*n* = 84). **Figure S2**. Distribution of delay in reporting among fever cases in community- and hospital-based surveys. **Figure S3**. Classification plots for each cut-off value using hospital-based survey. **Figure S4**. Calibration plots for each cut-off value using hospital-based survey.

## Data Availability

Data used and analysed during this study are available from the corresponding author only for research purpose which can be shared with the consent of the Indian Council of Medical Research.

## **Abbreviations**

ROC: Receiver Operating Characteristic
WHO: World Health Organization
NIMR: National Institute of Malaria Research
NVBDCP: National Vector Borne Disease Control Programme
RDT: Rapid Diagnostic Test
PSU: Primary Sampling Unit
AIC: Akaike information criterion
BIC: Bayesian information criterion
PPV: Positive Predictive Value
NPV: Negative Predictive Value
CITL: Calibration-In-The-Large
OR: Odds Ratio
CI: Confidence Interval
INR: Indian Rupees
aOR: Adjusted Odds Ratio
SD: Standard Deviation
km: Kilometre
