# Supplementary material for: Determination of cut-off and correlates of delay in treatment-seeking of febrile illness: a retrospective analysis
Source: BMC Public Health. 2020 Apr 28;20:572. doi: 10.1186/s12889-020-08660-2 (PMC7189459; doi:10.1186/s12889-020-08660-2)

**Supplementary materials**

Figure S1: Spearman rank correlation between delay in reporting fever and duration of fever treatment in community-based survey (n = 84)


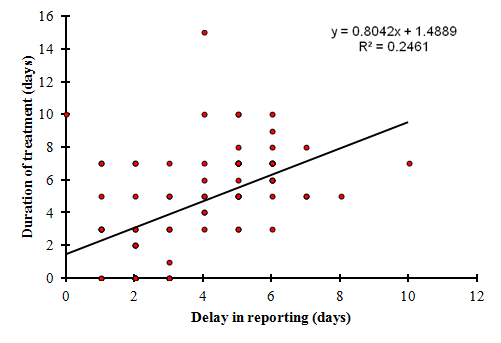


Figure S2: Distribution of delay in reporting among fever cases in community- and hospital-based surveys.


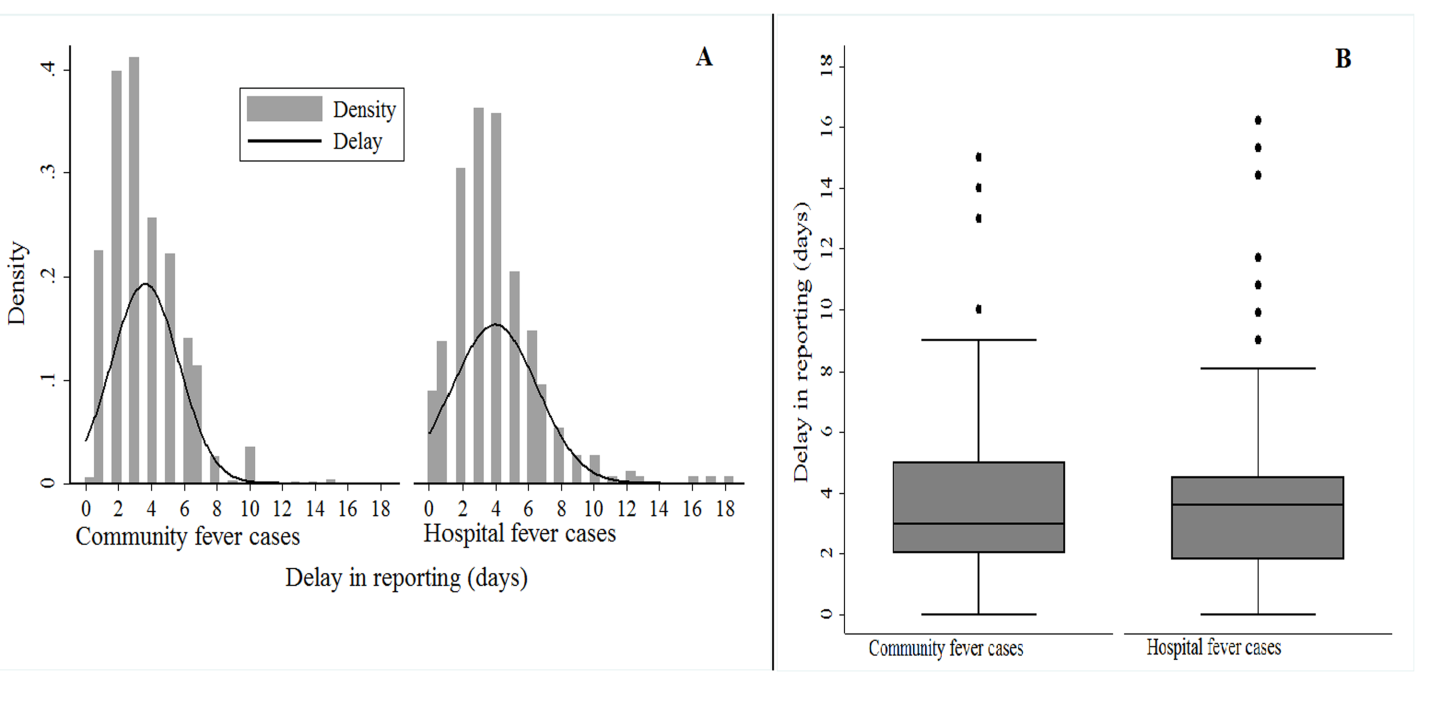


Figure S3: Classification plots for each cut-off value using hospital-based survey.

Figure S4: Calibration plots for each cut-off value using hospital-based survey.


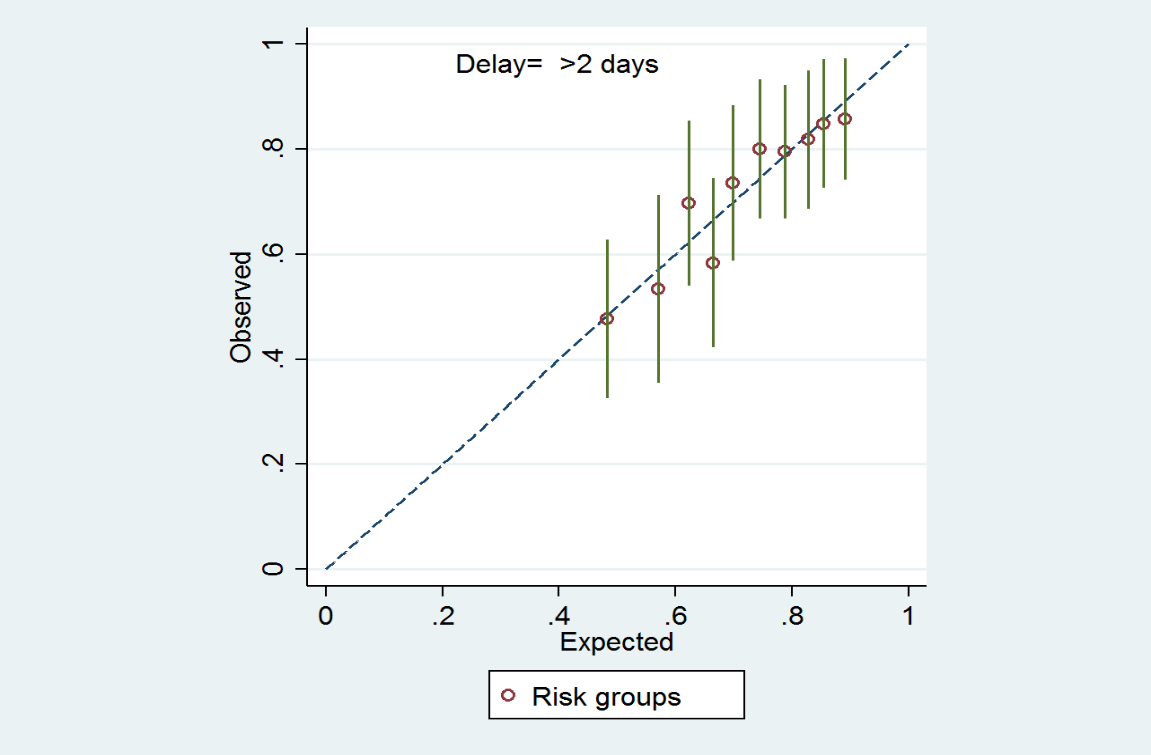

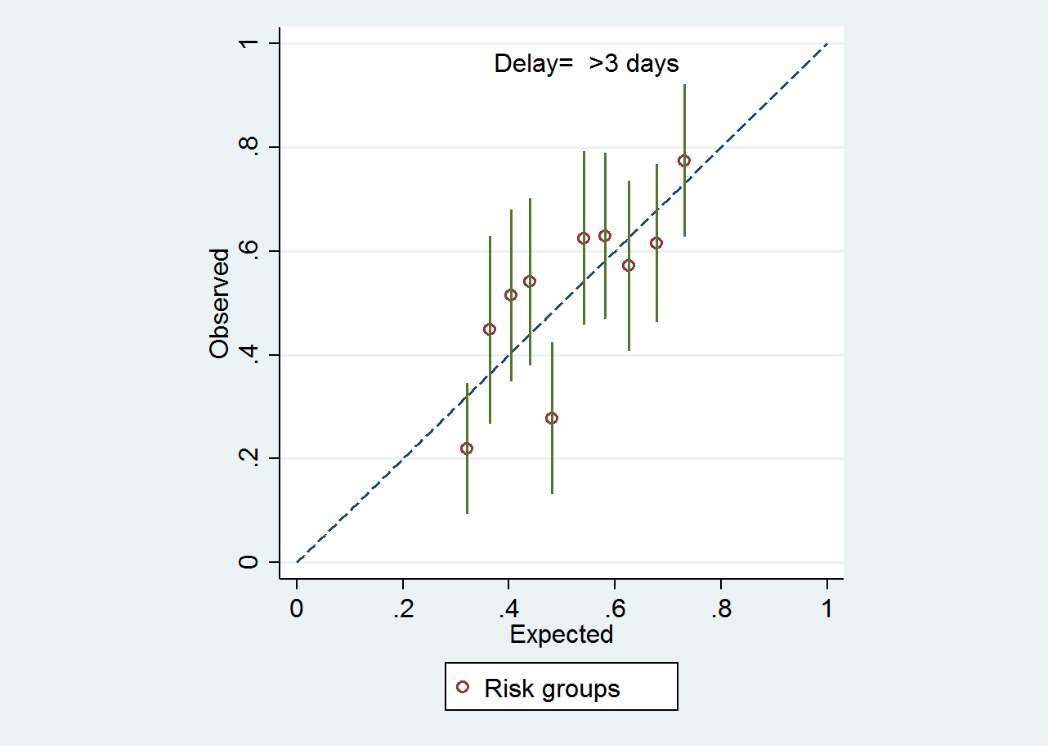


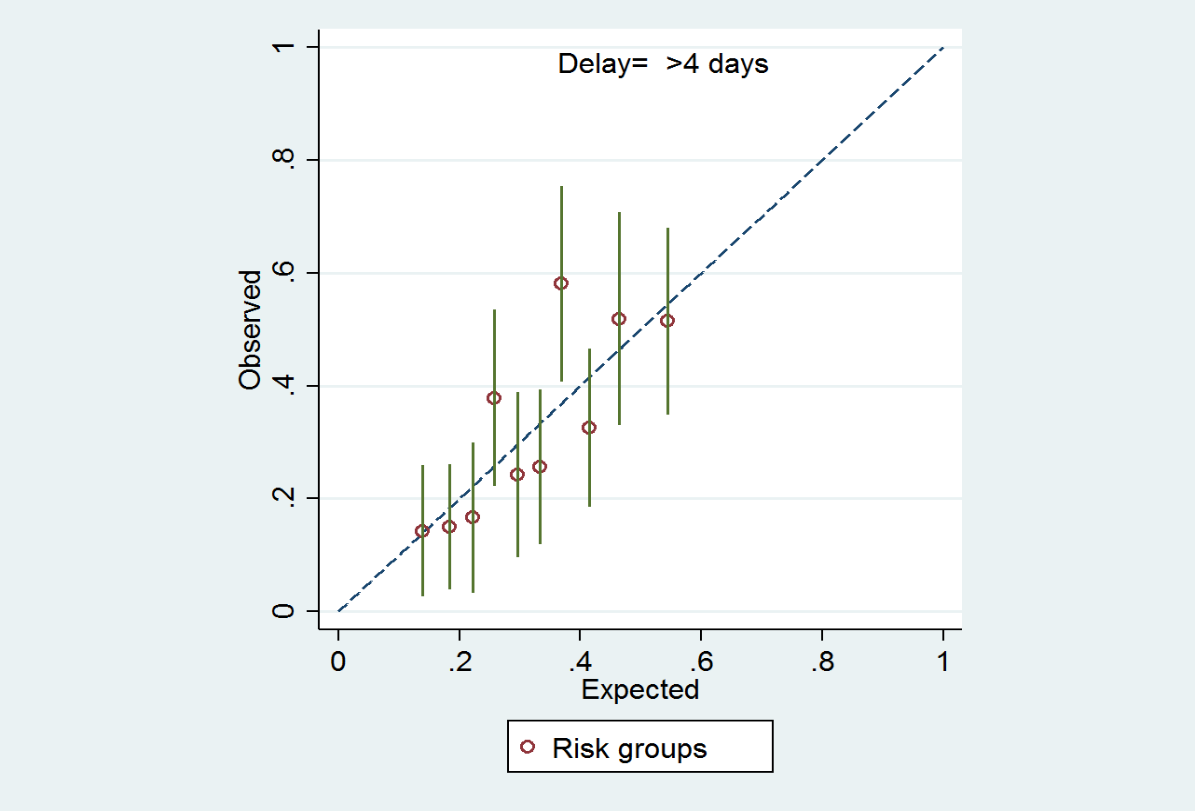

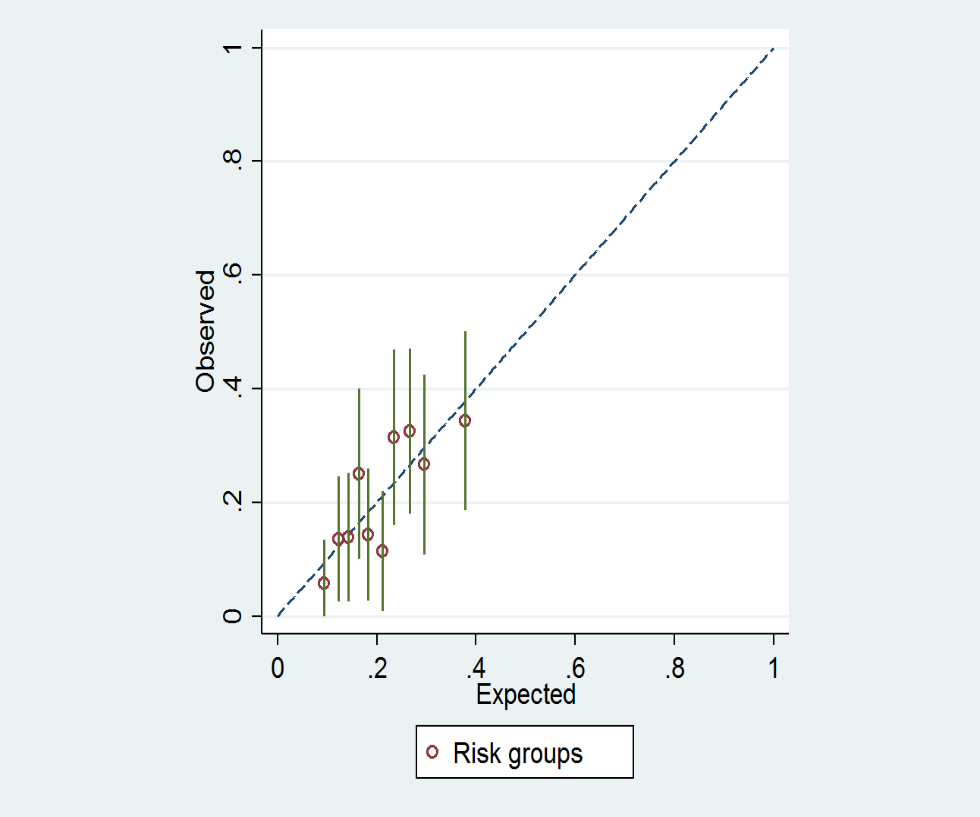

Supplement: Supplementary file 1 — Additional file 1 Figure S1. Spearman rank correlation between delay in reporting fever and duration of fever treatment in community-based survey (n = 84). Figure S2. Distribution of delay in reporting among fever cases in community- and hospital-based surveys. Figure S3. Classification plots for each cut-off value using hospital-based survey. Figure S4. Calibration plots for each cut-off value using hospital-based survey. [file 12889_2020_8660_MOESM1_ESM.docx]
